# An *in silico* guide for ventriculo-ventricular delay programming for left bundle branch-optimized cardiac resynchronization therapy

**DOI:** 10.1093/europace/euaf089

**Published:** 2025-05-21

**Authors:** Marina Strocchi, Jack W Samways, Akriti Naraen, Nadine Ali, Matthew J Shun-Shin, Karli Gillette, Christopher Aldo Rinaldi, Ahran D Arnold, Gernot Plank, Edward J Vigmond, Zachary I Whinnett, Steven A Niederer

**Affiliations:** National Heart and Lung Institute, Imperial College London, 72 Du Cane Road, London W12 0NN, UK; National Heart and Lung Institute, Imperial College London, 72 Du Cane Road, London W12 0NN, UK; National Heart and Lung Institute, Imperial College London, 72 Du Cane Road, London W12 0NN, UK; National Heart and Lung Institute, Imperial College London, 72 Du Cane Road, London W12 0NN, UK; National Heart and Lung Institute, Imperial College London, 72 Du Cane Road, London W12 0NN, UK; Gottfried Schatz Research Center, Division of Medical Physics and Biophysics, Medical University of Graz, Graz, Austria; Department of Biomedical Engineering, University of Utah, Salt Lake City, UT, USA; Scientific Computing and Imaging Institute, University of Utah, Salt Lake City, UT, USA; School of Biomedical Engineering and Imaging Sciences, King’s College London, London, UK; Cardiovascular Department, Guys’ and St Thomas’ NHS Foundation Trust, London, UK; National Heart and Lung Institute, Imperial College London, 72 Du Cane Road, London W12 0NN, UK; Gottfried Schatz Research Center, Division of Medical Physics and Biophysics, Medical University of Graz, Graz, Austria; BioTechMed-Graz, Graz, Austria; IHU Liryc, Electrophysiology and Heart Modeling Institute, Fondation University Bordeaux, Pessac-Bordeaux, France; Institute of Mathematics of Bordeaux, UMR 5251, University of Bordeaux, Bordeaux, Talence, France; National Heart and Lung Institute, Imperial College London, 72 Du Cane Road, London W12 0NN, UK; National Heart and Lung Institute, Imperial College London, 72 Du Cane Road, London W12 0NN, UK; The Alan Turing Institute, London, UK

**Keywords:** Cardiac resynchronization therapy, Ventriculo-ventricular delay, Conduction substrate, Conduction system pacing, Computational model

## Abstract

**Aims:**

Left bundle branch pacing (LBBP)–optimized cardiac resynchronization therapy (LOT-CRT) can improve left ventricular (LV) activation when LBBP alone or conventional biventricular pacing are ineffective. However, the optimal programming settings for ventriculo-ventricular delay (VVD) for LOT-CRT are unknown. We aim to investigate how to optimally program VVD for LOT-CRT in the presence of various LV conduction substrates using computational modelling.

**Methods and results:**

We simulated ventricular activation on 24 anatomies and validated the model against clinical data. Diffuse LV conduction system and intra-myocardial delay were simulated by slowing the conduction velocity of the LV His-Purkinje system and myocardium, respectively, alone or in combination with proximal left bundle branch block (LBBB). We simulated LOT-CRT with selective or myocardial capture (LV septal pacing, LVSP) with VVD ranging between −100 ms (LBBP/LVSP ahead) and +100 ms [LV epicardial lead (LVepiP), ahead]. Response was quantified with 95% LV activation times (LVAT95). In the presence of diffuse LV conduction system delay, the optimal VVD for LOT-CRT was always negative (LBBB: −42.5 ± 6.6 ms; no LBBB: −36.2 ± 5.6 ms), as delivering LBBP ahead of LVepiP compensates for the slow LV His-Purkinje. In the presence of LV intra-myocardial disease, the shortest LVAT95 with LOT-CRT was achieved by pacing the coronary sinus LV first (optimal VVD for LBBB: 23.3 ± 8.5 ms; no LBBB: 79.2 ± 18.0 ms). The type of capture for LOT-CRT affected the optimal VVD, with myocardial capture favouring negative VVDs (LVSP ahead).

**Conclusion:**

The optimal VVD for LOT-CRT depends on the mechanism of delayed LV activation and type of capture achieved, highlighting the importance of VVD optimization.

## Graphical Abstract

**Graphical Abstract:**
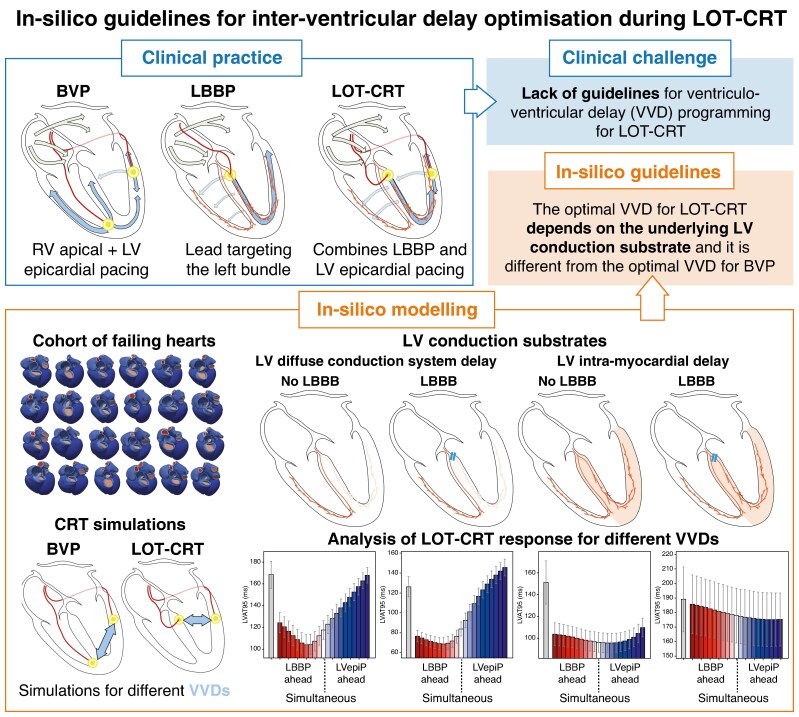

**What’s new?**

- Left bundle branch pacing (LBBP) alone is not effective in patients with left ventricular (LV) diffuse conduction disease
- LBBP–optimized cardiac resynchronization therapy (LOT-CRT) can improve LV activation in these patients, but response may be dependent on the selected ventriculo-ventricular delay (VVD)
- It is not known how to optimally program the VVD for LOT-CRT in the presence of different LV conduction substrates
- Our results show that the optimal VVD depends on the LV conduction substrate causing the LV dyssynchrony
- This study provides an *in silico* guide on how to optimally program the VVD in different patient groups

## Introduction

Cardiac resynchronization therapy (CRT) improves mortality and morbidity in patients with heart failure (HF) and prolonged QRS duration, particularly in the presence of left bundle branch block (LBBB).^1,2^ Cardiac resynchronization therapy is often delivered via biventricular pacing (BVP) by implanting pacing leads at the right ventricular (RV) endocardium and at the left ventricular (LV) epicardium [LV epicardial pacing (LVepiP)] in a coronary sinus tributary. In standard practice, BVP is delivered by pacing the LV and the RV simultaneously or with a short offset [ventriculo-ventricular delay (VVD)], as studies investigating VVD optimization for BVP have failed to consistently show significant advantages of VVD optimization.^3–5^ On the other hand, Wisnoskey *et al.*^6^ have shown that optimizing the atrioventricular delay (AVD) during BVP or LV pacing to achieve optimal fusion between pacing and intrinsic activation leads to better response to CRT, highlighting the potential of tailoring the device settings to a specific patient. Despite the clear benefits of BVP for the majority of the patients, many still do not respond to CRT^1^ and, even in those who do, BVP leads to a non-physiological activation sequence because it relies on slow myocyte-to-myocyte propagation.

Conduction system pacing (CSP) is an emerging alternative modality for CRT delivery, where either the bundle of His [His bundle pacing (HBP)] or the left bundle branch [left bundle branch pacing (LBBP)] are paced to engage the fast conduction system of the ventricles, either selectively by pacing only the His-Purkinje system, or non-selectively by pacing the surrounding myocardium as well.^7,8^ Both HBP and LBBP can completely correct proximal conduction system block (a block occurring along the His bundle) to restore physiological, synchronous LV activation^9–11^ but with a larger target zone and more reliable pacing parameters, LBBP or left bundle branch area pacing (LBBAP, when the left bundle and/or the LV septal myocardium is paced) is the favoured approach in many centres. Conduction system pacing constitutes an attractive alternative to BVP as in patients with proximal block in the His or left bundle; it can restore normal physiological LV activation and thereby results in more effective LV resynchronization compared with BVP. However, questions remain about the effect of LBBP on RV activation and synchrony, even though these effects may be attenuated by atrioventricular delay optimization.^10^ Furthermore, results with LBBP are less favourable when delivered to patients who have distal or diffuse delay that cannot be corrected by CSP.^12,13^

When CSP does not completely resynchronize the LV, HBP, or LBBP can be combined with a conventional epicardial LV lead in a hybrid approach to deliver His-optimized or left bundle branch-optimized CRT (HOT-CRT and LOT-CRT), respectively.^14,15^ Initial studies have shown that LOT-CRT leads to better electrical and echocardiographic response compared with BVP.^14,16^ As with conventional BVP, the LOT-CRT hybrid approach allows programming the VVD, in this case representing the offset between LBBP and LVepiP.^15^ Due to the inconsistent results produced by studies investigating VVD optimization for BVP, BVP is often delivered with near simultaneous pacing. However, there are no studies to date investigating the effect of VVD optimization in response to LOT-CRT, particularly in the presence of different LV conduction substrates, which are known to affect response to LBBP.^12,17^ This leaves many open questions about how to program the VVD for LOT-CRT:

- Does the optimal VVD for LOT-CRT depend on the ventricular substrate?
- Is the optimal VVD the same for BVP and LOT-CRT?
- Does the optimal VVD vary with the type of LBBAP capture achieved?

In this study, we use computational electrophysiology to address the questions above. We validated the baseline model against electrocardiographic imaging (ECGi) data during LBBB, BVP, and CSP. We then simulated BVP, LBBAP, and LOT-CRT with different VVDs in the presence of different LV conduction substrates to provide a comprehensive *in silico* guidance for how to program the VVD during LOT-CRT in different patient groups.

## Methods

**Figure 1:**
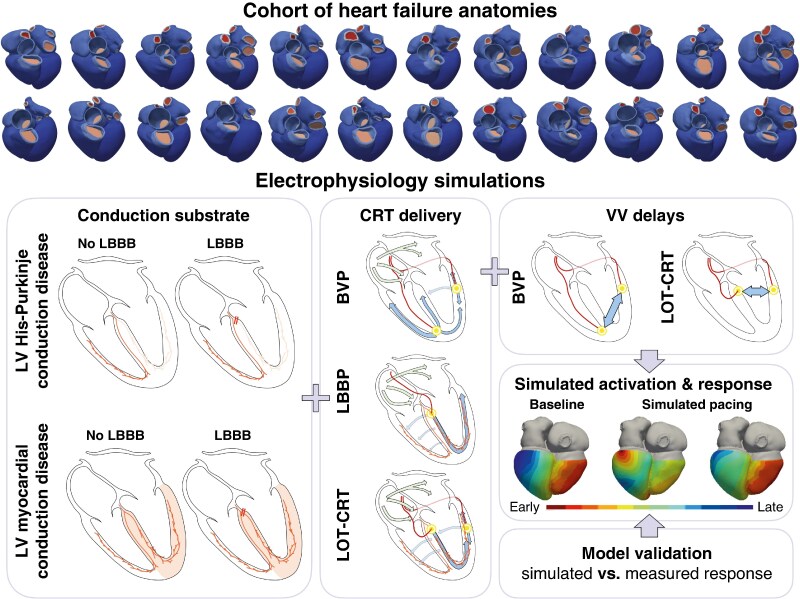
*In silico* study pipeline. Twenty-four publicly available heart failure anatomies were used in this study (top). Two LV conduction substrates were simulated: LV diffuse conduction system delay and LV intra-myocardial delay, in the presence or in the absence of proximal LBBB. We simulated CRT delivery methods (BVP, LBBP, and LOT-CRT), and BVP and LOT-CRT were simulated with different VVDs. We validated the model against clinically measured response, and we quantified LV synchrony with different VVDs for different LV conduction substrates. BVP, biventricular pacing; CRT, cardiac resynchronization therapy; LBBB, left bundle branch pacing; LOT-CRT, left bundle branch pacing–optimized cardiac resynchronization therapy; LV, left ventricular; VVD, ventriculo-ventricular delay.

A publicly available cohort of 24 whole-heart meshes generated from CT datasets collected from HF patients was used (*Figure 1*, top).^18^ Details on the computational modelling techniques used in this study were published previously.^12,19,20^ Briefly, a His-Purkinje system was generated, accounting for the presence of three LV fascicles (anterior, posterior, and septal) and two RV fascicles (septal and moderator band).^21^ Electrical activation of the ventricles was simulated with a reaction-Eikonal model,^22^ which computes local activation times provided initial stimuli locations and timings, and conduction velocities (CVs) in the local myofibre, sheet and normal-to-sheet directions. Conduction velocity refers to the propagation speed of a tissue type within the heart. The simulations in this study account for different propagation speeds (or CV) for the LV and RV myocardium and the ventricular fast conducting system, including the three LV fascicles (anterior, posterior, and septal), the two RV fascicles (septal and moderator band), and the LV and the RV Purkinje networks. For our baseline simulations, the myocardium was modelled as a transversely isotropic conduction material with a faster CV in the local myofibre direction of 0.6 m/s and anisotropy ratio of 0.4, consistently with measurements performed in mammals.^23^ The His-Purkinje system was assigned with an isotropic CV of 3 m/s.^24^ The CV of the individual fascicles (three in the LV and two in the RV) was computed so that, during sinus rhythm, the activation wave reached the end of all three LV fascicles at the same time and 10 ms before the end of the two RV fascicles, to be consistent with the Durrer maps.^25^

### Model validation

Before using the model to make considerations about response to LOT-CRT in the presence of different LV conduction substrates, we validated the model against available clinical data collected from a different cohort of 23 LBBB patients undergoing a CRT study, to ensure that the model replicates baseline LBBB activation metrics as well as clinically measured electrical response to different pacing modalities. The clinical data used for the validation have been published previously,^9^ and we provide details about it in Supplementary material online, *Section 1*. Briefly, 23 LBBB patients were enrolled in an ECGi study. Local ventricular activation times were computed from the epicardial voltage maps collected during baseline LBBB, BVP, HBP, and LBBP. We replicated this study by simulating proximal LBBB (baseline), BVP, by pacing the RV apex and the LV epicardium at the latest activated area simultaneously (VVD of 0 ms), HBP and LBBP, by selectively pacing the His and the left bundle, respectively. Because ECGi data only account for the activation of the epicardial surface of the heart, we extracted the epicardium of the ventricles from our 24 anatomical models and used the simulated epicardial activation to compute electrical response to compare to the data. We compared baseline total ventricular and LV activation times (TAT and LVTAT), and response to pacing as the reduction in the shortest time interval taken to activate 95% of the ventricles and of the LV (BIVAT95 and LVAT95). To compare the clinical measurements and the simulations, we established whether the variables were normally distributed with a Shapiro–Wilk test with a level of significance of 0.05. We then used a two-sided *t*-test or a Mann–Whitney *U* test if the variables were normally distributed or not, respectively. We corrected for multiple comparisons (*N* = 10) using a Bonferroni correction.

### Simulating different left ventricular conduction diseases

By mapping LV septum in LBBB patients, Upadhay *et al*.^17^ identified three different types of LV conduction diseases causing a LBBB-like morphology on a 12-lead ECG: proximal LBBB, diffuse (or distal) LV conduction system delay, and intra-myocardial delay. We replicated these in our model as follows:

1. *Proximal (or intrahisian) LBBB:* introduced by cutting the connection between the left bundle and the LV Purkinje network along the His
2. *Diffuse (or distal) left conduction system delay:* simulated by slowing the propagation speed of the LV His-Purkinje system
3. *Intra-myocardial delay:* simulated by slowing the propagation speed of the LV myocardium

Because these LV conduction diseases can happen alone or in combination, we considered the following scenarios:

- *Scenario A:* (1) and (2) in combination to simulate a proximal block, corrected by LBBP, concomitant with LV diffuse conduction system delay not correctable with LBBP
- *Scenario B:* (1) and (3) in combination to simulate a proximal block, corrected by LBBP, concomitant with LV intra-myocardial delay, not correctable with LBBP
- *Scenario C:* (2) alone (with healthy proximal His-Purkinje system) to simulate prolonged LV activation caused purely by LV diffuse conduction system disease, not corrected by LBBP
- *Scenario D:* (3) alone (with healthy proximal His-Purkinje system) to simulate prolonged LV activation caused purely by LV intra-myocardial disease, not corrected by LBBP

For these scenarios, we simulated two different degrees of LV conduction system delay and LV intra-myocardial delay to investigate the effect of conduction slowing severity on our results. For scenario A and B, mild and severe LV diffuse conduction system delay and intra-myocardial delay were simulated by setting the CV of the LV His-Purkinje or the LV myocardium to 60% and 40% of healthy CV, respectively. In the absence of proximal LBBB (scenarios C and D), 60% of healthy CV did not lead to significantly prolonged LV activation times. In this case, we simulated mild and severe LV diffuse conduction system delay and LV intra-myocardial delay by setting the CV of the LV His-Purkinje or the LV myocardium to 40% and 20% of healthy CV, respectively.

### Pacing simulations

Selective LBBP (S-LBBP) was simulated by pacing the left bundle below the level of proximal LBBB. Because achieving conduction system capture can be challenging in clinical practice, we also simulated non-selective LBBP (NS-LBBP) and LV septal myocardial capture (LV septal pacing, LVSP), by pacing the myocardium around the LBBP site with or without the left bundle, respectively, to investigate the effect of the type of capture on our results. An LV epicardial stimulus in the latest activated area during baseline was introduced to simulate LVepiP. Left bundle branch pacing–optimized cardiac resynchronization therapy and BVP were then simulated by performing LBBAP, and RV apical pacing in combination with LVepiP. We simulated LOT-CRT with selective (S-LBBP + LVepiP), non-selective (NS-LBBP + LVepiP), and purely myocardial (LVSP + LVepiP) capture and BVP with different VVD, ranging from −100 ms to +100 ms in 10 ms steps. Negative and positive VVD indicates LBBAP/RV pacing ahead and LVepiP ahead, respectively. All simulations were carried out under the assumption that pacing overrides the patient’s intrinsic activation.

Left bundle branch pacing–optimized cardiac resynchronization therapy is a pacing modality mainly aimed at shortening LV activation times. Therefore, the response was quantified in terms of reduction of the shortest time interval taken to activate 95% of the LV (LVAT95) from baseline. When computing response, the area around the atrioventricular valves was excluded, and the intraventricular septum was divided evenly between the LV and the RV transmurally.

### Questions addressed

We used the pacing simulations described above in combination with scenarios A-D simulating different LV conduction diseases to answer the following questions about VVD optimization for LOT-CRT:

- *Does the optimal VVD for LOT-CRT depend on the ventricular conduction substrate?* We simulated LOT-CRT with selective LBBP with different VVDs for scenarios A-D, representing different LV conduction substrates. We then compared the LVAT95 to identify the optimal VVD on average across the patient group for each diseased scenario.
- *Is the optimal VVD the same for BVP and LOT-CRT?* We simulated BVP and LOT-CRT with selective LBBP with different VVDs for scenarios A and B (proximal LBBB combined with LV diffuse conduction system disease or intra-myocardial delay). LVAT95 was then computed for each VVD to compare the optimal average delay for BVP and LOT-CRT.
- *Does the optimal VVD vary with the type of LBBAP capture achieved?* We simulated LOT-CRT with non-selective LBBP and with LVSP (purely LV septal myocardial capture) with different VVD for scenarios A and B. We then identified and compared the optimal average VVD for the patient cohort for non-selective and myocardial capture.

## Results

First, we show a validation of the model during baseline LBBB, BVP, and LBBP, to demonstrate that the simulations replicate *in vivo* clinical observations. Then, we use the model to address the questions above by simulating: LOT-CRT with varying VVDs for different conduction substrates; LOT-CRT and BVP with varying VVDs; LOT-CRT with different types of LBBAP capture with varying VVDs. For each scenario, the optimal VVD on average across the cohort was compared between modalities to build an *in silico* guide on VVD programming for different conduction disturbances.

### Model validation

**Figure 2:**
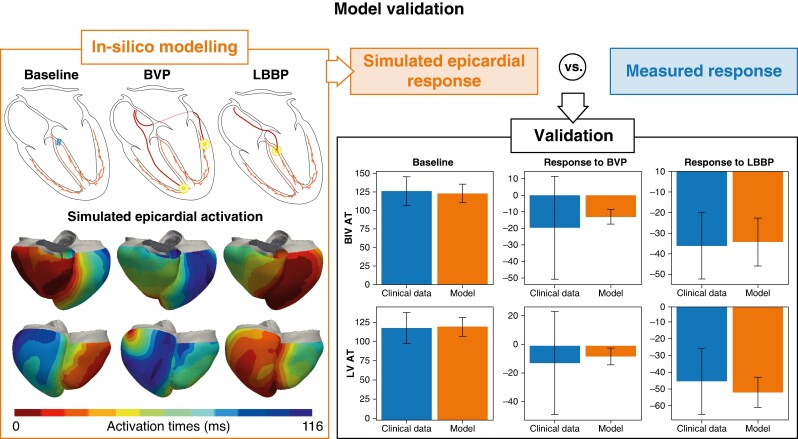
The *in silico* model was validated against response from electrocardiographic (ECGi) data during baseline (proximal LBBB), biventricular pacing (BVP) and left bundle branch pacing (LBBP). On the left, we show a schematic of the modelled scenarios with the corresponding simulated epicardial activation maps. On the right, we compare the simulated and the measured electrical epicardial response by plotting the mean biventricular and LV activation time during baseline, and their reduction in response to BVP and LBBP. The black lines show ± 1 SD, and blue and orange bars show the measured and the simulated data, respectively. LV, left ventricular.

We ensured that the model reliably replicated clinical observations by comparing the predicted activation metrics during baseline, BVP and LBBP (*Figure 2*). The simulated TAT and LVTAT during baseline (proximal LBBB) were consistent with the clinically measured metrics (TAT: epicardial model 122.7 ± 12.4 ms vs. clinical 126.1 ± 19.4 ms, *P* = 0.7279; LVTAT: epicardial model 121.2 ± 12.3 ms vs. clinical 119.5 ± 20.2 ms, *P* = 0.2234).

During BVP, the model predicted a BIVAT95 shortening of −13.1 ± 4.4 ms, comparable to the clinically measured response of −19.7 ± 31.1 ms (*P* = 0.4791). Similarly, the predicted and clinically measured reduction in LVAT95 following BVP were similar (−7.4 ± 5.8 vs. −12.0 ± 36.1 ms, *P* = 0.6705). Simulated biventricular response to LBBP was −34.3 ± 11.6 ms, comparable to the clinically measured ventricular response of −36.2 ± 16.2 ms (*P* = 0.6763). Simulated and measured LV response to LBBP were also similar (−51.1 ± 9.1 ms vs. −44.5 ± 19.8 ms, *P* = 0.1796). This validation shows that the baseline model during proximal LBBB can replicate measured ventricular and LV activation times. Furthermore, the predicted reduction in epicardial BIVAT95 and LVAT95 during BVP and LBBP matched the *in vivo* response measured with ECGi. This demonstrates that the model reliably predicts response to the pacing modalities of interest and can therefore be used to make observations about response to LOT-CRT.

### The optimal VVD for LOT-CRT depends on the conduction substrate

**Figure 3:**
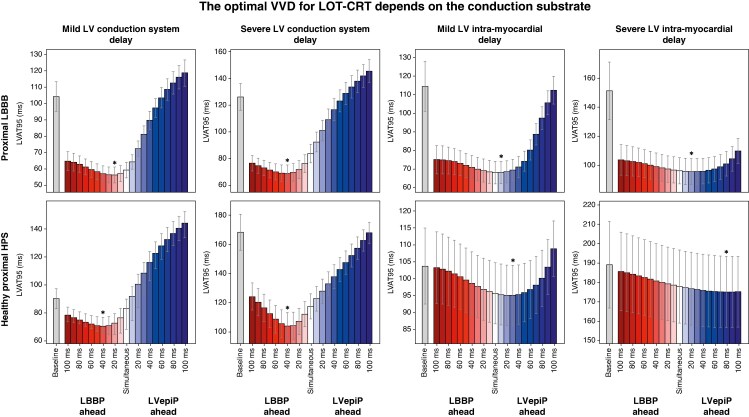
Response to LOT-CRT for different VVDs for different LV conduction substrates. The top row shows the LVAT95 for proximal LBBB combined with different LV conduction substrates. From left to right: mild and severe LV diffuse conduction system delay, simulated as LV His-Purkinje system CV slowed to 60% and 40% of healthy CV; mild and severe LV intra-myocardial delay, simulated as LV myocardium CV slowed to 60% and 40% of healthy CV. The bottom row shows the same results with LV conduction substrates alone, without proximal LBBB (with healthy proximal His-Purkinje system, HPS), where mild and severe conduction diseases were simulated using 40% and 20% of healthy CV, respectively. All results are shown for VVDs ranging from −100 ms (LBBP ahead) to +100 ms (LVepiP ahead). The grey bars and the stars indicate the LVAT95 at baseline and the best VVD on average, respectively, while the error bars indicate ± the standard deviation. CV, conduction velocity; HPS, His-Purkinje system; LBBB, left bundle branch block; LBBP, left bundle branch pacing; LOT-CRT, left bundle branch pacing–optimized cardiac resynchronization therapy; VVD, ventriculo-ventricular delay.

To address the first question and investigate whether the optimal VVD for LOT-CRT depends on the LV conduction substrate, we simulated LOT-CRT for scenarios A-D representing proximal LBBB combined with LV diffuse conduction system or intra-myocardial delay, and LV conduction system or intra-myocardial delay with healthy proximal His-Purkinje system. *Figure 3* shows how LVAT95 simulated during LOT-CRT changes in response to positive (LVepiP ahead) and negative (LBBP ahead) VVDs in the presence of different types and severities of LV conduction diseases, with (top) or without (bottom) proximal LBBB. In the presence of LV diffuse conduction system delay, the optimal VVD is always negative (LBBB + severe slowing: −42.5 ± 6.6 ms; no LBBB + severe slowing: −36.2 ± 5.6 ms), indicating that LBBP should be performed ahead of LVepiP. Pacing the LBBP ahead compensates for the slow LV His-Purkinje and leads to shorter LVAT95, with more negative delays required for more severe conduction disease. On the other hand, in the presence of LV intra-myocardial delay, the shortest LVAT95 is achieved by pacing the LVepiP ahead (optimal VVD for LBBB + severe slowing: 23.3 ± 8.5 ms; no LBBB + severe slowing: 79.2 ± 18.0 ms). In this case, the positive VVD compensates for the slow myocardium and more positive delays are required for more severe conduction disease. These results suggest that the optimal VVD during LOT-CRT is dependent on the diffuse conduction disease causing prolonged LV activation times (either slow propagation of the conduction system or myocardium), showing the importance of VVD personalization during LOT-CRT.

In some patients, slow LV myocardial conduction is regional rather than uniform due to the presence of scar. In the supplement, we show that the optimal VVD for LOT-CRT changes not only due to the presence of scar in the LV lateral wall, but also in response to the conduction properties of the scarred tissue.

HOT-CRT is an alternative pacing method to LOT-CRT combining HBP and LV epicardial pacing to shorten LV activation times in patients with residual LV activation delay following conduction system pacing. To test whether the optimal VVD for HOT-CRT was different compared with LOT-CRT, we performed simulations with VVDs between −100 ms and +100 ms with HOT-CRT. In the supplement, we demonstrate that the optimal VVD for LOT-CRT and HOT-CRT are similar for different conduction disturbances, making our results valid to HOT-CRT as well as LOT-CRT.

### Optimal response to BVP and LOT-CRT is achieved with different VVDs

**Figure 4:**
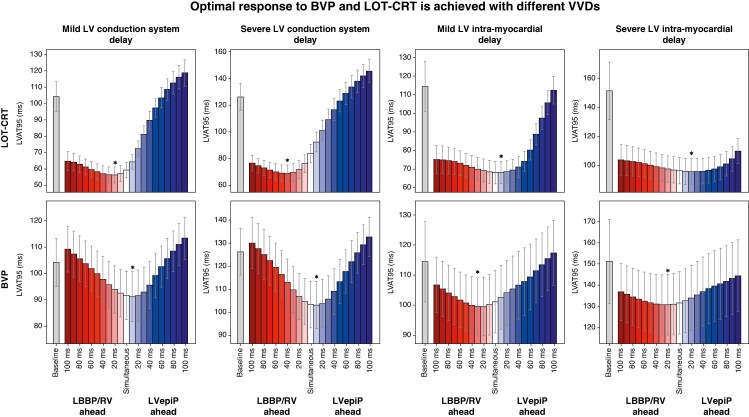
Comparison between LOT-CRT and BVP for different VVDs and different LV conduction diseases. Mean LVAT95 is shown as a function of VVDs during LOT-CRT (top row) and BVP (bottom row). Different columns represent different conduction substrates in combination with proximal LBBB. From left to right: mild and severe LV conduction system delay, simulated as LV His-Purkinje system CV slowed to 60% and 40% of healthy CV; mild and severe LV intra-myocardial delay, simulated as LV myocardium CV slowed to 60% and 40% of healthy CV. All results are shown for VVDs ranging from −100 ms (LBBP or RV pacing ahead) to +100 ms (LVepiP ahead). The grey bars and the stars indicate the LVAT95 at baseline and the best VVD on average, respectively, while the error bars indicate ± the standard deviation. CV, conduction velocity; LBBB, left bundle branch block; LBBP, left bundle branch pacing; LOT-CRT, left bundle branch pacing–optimized cardiac resynchronization therapy; VVD, ventriculo-ventricular delay.

To address the second question and assess whether the optimal VVD changes between LOT-CRT and BVP, we compared the optimal VVD for BVP and LOT-CRT in the presence of proximal LBBB combined with LV diffuse conduction system (scenario A) or with LV intra-myocardial delay (scenario B). *Figure 4* shows LVAT95 during LOT-CRT (top row) and BVP (bottom row) simulated with varying VVDs in these scenarios. The variation between optimal VVDs for BVP with different electrical substrates is smaller than for LOT-CRT, and the optimal VVDs are close to simultaneous offset. For example, with mild LV diffuse conduction system disease (*Figure 4*, first column), different VVDs caused the LVAT95 to vary between 56.1 ± 4.9 ms and 118.6 ± 8.1 ms, and between 91.2 ± 9.5 ms and 113.4 ± 8.0 ms with LOT-CRT and BVP, respectively. With LV diffuse conduction system delay, the optimal VVD for BVP is positive but near simultaneous (severe: 7.1 ± 8.4 ms), whereas for LOT-CRT the optimal VVD is negative (severe: −42.5 ± 6.6 ms). With LV intra-myocardial delay, a slight negative VVD is optimal for BVP (severe: −9.2 ± 37.3 ms), but a positive VVD is optimal for LOT-CRT (severe: 23.3 ± 8.5 ms). Our results also show that LOT-CRT leads to shorter LVAT95 compared with BVP for all LV conduction disturbances we considered. In the presence of severe LV diffuse conduction system disease, the shortest LVAT95 was 103.0 ± 10.3 ms, compared with 68.7 ± 6.3 ms with LOT-CRT. Similarly, in the presence of severe LV intra-myocardial delay, LOT-CRT and BVP led to LVAT95 of 95.6 ± 8.9 ms and 130.7 ± 14.1 ms, respectively. In the supplement, we show that, in the presence of LBBB without any additional LV conduction disturbances, LV epicardial pacing confers marginal additional LVAT95 shortening to LBBP alone. This indicates that, in patients with proximal conduction block, LBBP can normalize LV activation and that LOT-CRT may not be necessary.

### Different types of LBBAP capture during LOT-CRT affect the optimal VVD

**Figure 5:**
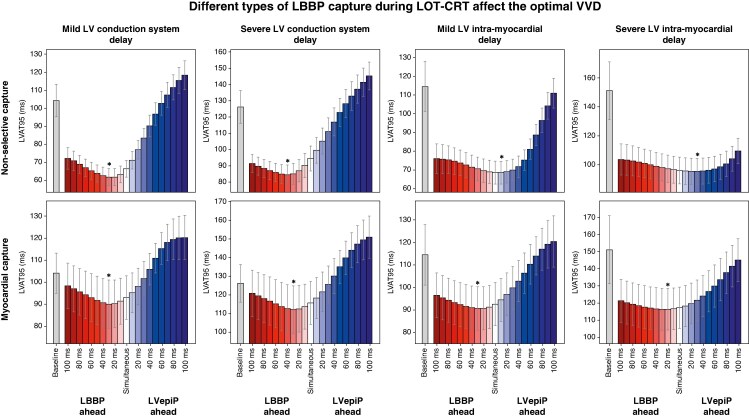
Comparison between response to LOT-CRT with non-selective and myocardial capture for different VVDs and different conduction substrates. Mean LVAT95 is shown as a function of VVDs during LOT-CRT with non-selective (top row) and purely myocardial (bottom row) capture (LV septal pacing). Different columns represent different LV conduction substrates in combination with LBBB. From left to right: mild and severe LV conduction system delay, simulated as LV His-Purkinje system CV slowed to 60% and 40% of healthy CV; mild and severe LV intra-myocardial delay, simulated as LV myocardium CV slowed to 60% and 40% of healthy CV. All results are shown for VVDs ranging from −100 ms (LBBP ahead) to +100 ms (LVepiP ahead). The grey bars and the stars indicate the LVAT95 at baseline and the best VVD on average, respectively, while the error bars indicate ± the standard deviation. CV, conduction velocity; LBBB, left bundle branch block; LBBP, left bundle branch pacing; LOT-CRT, left bundle branch pacing–optimized cardiac resynchronization therapy; VVD, ventriculo-ventricular delay.

To address the third question, we quantified the effect of the type of LBBAP capture during LOT-CRT on the optimal VVD by simulating LOT-CRT with non-selective LBBP and with purely myocardial capture (LVSP) for scenario A and B (proximal LBBB combined with LV diffuse conduction system or intra-myocardial delay). *Figure 5* shows LVAT95 simulated for these scenarios with mild or severe LV diffuse conduction system and intra-myocardial delay during non-selective LOT-CRT and LVSP for different VVDs. The optimal VVDs between selective and non-selective capture were similar (severe diffuse conduction system disease, selective: −42.5 ± 6.6 ms, non-selective: −40.8 ± 8.1 ms; severe intra-myocardial delay, selective: 23.3 ± 8.5 ms, non-selective: 23.8 ± 8.6 ms). Regardless of the LV conduction disease, LVSP led to longer LVAT95 compared with non-selective LOT-CRT. While the optimal VVD required to achieve the shortest LVAT95 with non-selective LOT-CRT changes depending on the type and severity of LV electrical substrate, optimal VVD for LOT-CRT with LVSP is always negative (LBBP ahead), regardless of the type of diffuse conduction disease we simulated (*Figure 5*, bottom). In the presence of LV diffuse conduction system delay, pacing the LBBAP ahead leads to shortest LVAT95, similar to LOT-CRT with non-selective capture (optimal VVD for severe conduction system delay with non-selective capture: −40.8 ± 8.1 ms; myocardial capture: −27.1 ± 14.6 ms). With LV intra-myocardial delay, we again found that programming LV septal pacing to occur prior to LV coronary sinus pacing provided the most rapid ventricular activation (optimal VVD for severe LV myocardial slowing with myocardial capture: −28.3 ± 17.5 ms). This shows that, if conduction system capture cannot be achieved during LOT-CRT, the optimal VVD might be less dependent on underlying substrate.

Clinical^26^ and computational^10^ studies have shown that LBBP can result in prolonged RV activation times, while LV activation times are short and comparable to HBP. However, prolonged RV activation can be improved with optimized AVD in patients with intact right bundle branch conduction. In the supplement, we show that performing LOT-CRT with AVD optimization only affects RV activation and that the optimal VVD to achieve the shortest LVAT95 was the same between LOT-CRT performed with and without AVD optimization.

## Discussion

The advent of CSP as a CRT modality required better understanding of the origin of QRS prolongation in patients with 12-lead ECG evidence of LBBB. Through LV septal mapping during CSP, Upadhyay *et al*.^17^ established that LBBB-like QRS morphologies can be produced by discrete block in the proximal left conduction system, diffuse or distal block in the left conduction system or intra-myocardial delay with intact left conduction system. The latter two electrical substrates cannot be resynchronized fully by CSP attempts to capture the left bundle and even when proximal block occurs, if in the presence of a mixed pattern of disease (proximal block combined with diffuse or intra-myocardial disease), the LV delayed activation is not always reversed by CSP. Residual LV delay can be addressed by combining CSP leads with a conventional coronary sinus branch LV leads to produce HOT/LOT-CRT. However, how to optimise the VVD for this hybrid approach in the presence of different conduction substrates remains an open clinical question.

In this study, we used validated computational modelling to provide in-silico guidance for LOT-CRT VVD optimization in the presence of different LV conduction substrates. We addressed three questions:

- Does the optimal VVD for LOT-CRT depend on the ventricular conduction substrate?
- Is the optimal VVD the same for BVP and LOT-CRT?
- Does the optimal VVD vary with the type of LBBAP capture achieved?

We found that, irrespective of whether the left bundle is captured or not, pacing the left bundle area (LBBAP) alongside LVepiP requires the LBBP lead to be paced 20–40 ms earlier than the LV lead for optimal VVD when there is any delay in the conduction system with subsequently healthy myocardial propagation, as shown in the first two columns of *Figures 3* and *5*. This allows the delayed onset, but physiological, myocardial activation from LBBAP to be preserved with minimal disruption from non-physiological myocardial pacing from the LV lead while still utilising it to correct the residual LV delay. Conversely, when intra-myocardial delay is responsible for QRS prolongation (third and fourth columns of *Figure 3*), the shortest LVAT is achieved by pacing the LV lead earlier than the LBBAP lead, with longer offsets needed for slower myocardial propagation. These results suggest that VVD optimization could be beneficial for LOT-CRT.

Some clinical studies reported significant benefits for VVD optimization for BVP, while others showed no significant differences compared with standard simultaneous pacing.^3–5,27^ Our findings offer a potential explanation for these inconsistencies, showing that the magnitude of LVAT response to varying VVD for BVP is not as large as for LOT-CRT (*Figure 4*) and that the optimal LVAT is relatively constant with LVepiP performed simultaneously or slightly ahead of RV pacing in the presence of LV intra-myocardial delay, which mirrors programming in routine clinical practice. Our findings suggest that this BVP practice cannot be translated to LOT-CRT, where a simultaneous offset is rarely the preferred setting.

Three capture types are exhibited when attempting LBBAP^28^: (i) selective LBBP where the left bundle is captured alone (rarely achieved at programmed outputs), (ii) non-selective LBBP where the left bundle is captured alongside the surrounding myocardium, and (iii) left ventricular septal pacing (LVSP) where only the myocardium is captured. Although clinical studies have not directly compared electrical synchrony achieved with selective vs. non-selective LBBP, only small qualitative differences in paced QRS morphologies have been reported, mainly due to RV septum pre-excitation through non-selective capture. In agreement with these results, we have previously shown non-significant differences between simulated electrical response between selective and non-selective LBBP using computational electrophysiology, while LVSP significantly increased paced activation times.^19^ Similarly, clinical studies have reported longer paced QRS durations during LVSP compared with LBBP.^29^ Nevertheless, LVSP has advantages over LBBP as it allows for shorter procedure times, and it can be achieved over a larger area of the septum, making it accessible for non-specialized centres.^29^ In this study, we simulated LBBP and LVSP with different VVDs to investigate the effect of capture on the optimal VVD. We have shown that, when intra-myocardial delay is responsible for QRS prolongation, the type of capture strongly influences the optimal VVD to program (third and fourth columns in *Figure 5*). This difference relates to the variation in septal activation time from LVSP and LBBP, as the LV lead must be timed to coincide with septal activation for synchrony. When diffuse conduction system delay is present (first and second column in *Figure 5*), both LVSP and LBBP favour early LBBAP as the septum is activated similarly (late) by both forms of LBBAP. These findings provide *in silico* guidance for VVD programming for LOT-CRT in patients and centres where only LVSP and not LBBP can be achieved or performed.

Our findings, summarized in *Table 1*, may be important for clinical practice in two ways. They support a role for personalized VVD optimization for LOT-CRT patients. When possible, an optimization procedure should be performed to determine the optimal VVD for an individual patient by, for instance, testing different VVDs during the implantation procedure and selecting the VVD achieving the best electrocardiographic, echocardiographic, or haemodynamic response,^30^ since a ‘nominal’ VVD cannot be determined for all LOT-CRT patients. Alternatively, VVDs must be estimated from the mechanism causing QRS prolongation, but this is challenging to assess with a standard 12-lead ECG. Further research will elucidate suitable methods for this, which could include ultra-high frequency ECG, ECGi,^9,31^ MRI, or multi-modality techniques that may allow non-invasive determination of the patient-specific conduction substrate to then guide optimal VVD setting. However, these methods can be complex and expensive. Where VVD optimization is not practically possible, our findings offer a guide for empirical VVDs, in particular, clinical scenarios. In the common scenario of successful capture of the left bundle with residual intra-myocardial delay, which can be recognized by a long time to R wave peak time in lead V6 despite an observed threshold transition from non-selective to selective capture, LOT-CRT is likely to be a clinically utilized approach. Our modelling proposes that the coronary sinus lead should activate earlier than the LBBP lead by 20–40 ms (*Figure 3*). Alternatively, when only LVSP is achieved without capture of the left bundle, the LBBAP lead should be programmed to activate earlier than the coronary sinus branch lead by about 30 ms (*Figure 5*). To translate these findings in routine clinical practice, accurate 12-lead ECG criteria for diagnosing electrical substrate type are needed.

**Table 1:** Summary of our results

Programming guidance for LOT-CRT		
	Diffuse conduction disease	Intra-myocardial delay
**LBBP (selective or non-selective)**	Program VVD with *LBBP first* (∼20–40 ms)	Program VVD with *LV epicardial pacing first* (∼20–40 ms, longer VVDs required for more severe delays)
**LV septal pacing**	Programming VVD with LVSP ahead (∼30 ms) likely to produce most rapid LV activation	

The table summarizes the results from our modelling study. The rows indicate different types of capture (selective or non-selective LBBP vs. LV septal pacing, with purely myocardial capture), and the columns indicate different conduction diseases. Diffuse conduction disease indicates slowing of the LV His-Purkinje system, while intra-myocardial delay represents LV myocardial slowing.

LBBP, left bundle branch pacing; LOT-CRT, left bundle branch pacing–optimized cardiac resynchronization therapy; LV, left ventricular; LVSP, LV septal pacing; VVD, ventriculo-ventricular delay.

### Limitations

*In silico* trials allow systematic comparison of pathologies and therapies. However, they rely on models and have inherent limitations. Our study assumes that acute pacing-induced electrical synchrony correlates with long-term functional response, while additional factors other than electrical synchrony contribute to patient outcome. Nevertheless, a systematic review of CRT clinical trials has reported that responders had significantly larger QRS narrowing compared with non-responders,^32^ highlighting the importance of electrical synchrony following CRT. While this study did not include clinical endpoints to establish the long-term effects of VVD optimization, our results provide a template for prospective studies exploring clinical endpoints and outcomes. Our results accounted only for electrical synchrony and not for mechanical synchrony. Finite elements mechanics simulations currently suffer from high computational costs, in the order of hours on a supercomputer. Therefore, performing an *in silico* trial with multiple patients with many different pacing scenarios and settings like the one presented in this study would not be feasible. Circulatory system models represent an efficient alternative to whole-heart mechanics models and have been used in the past to find new biomarkers for long-term response.^33–36^. In future, combining our model with whole-heart mechanics or circulatory system models will allow us to extend these results to account for mechanical synchrony.

Our heart models include a synthetically generated His-Purkinje systems, which were not adapted to represent a specific patient. Nevertheless, the model validation we reported in the Supplement shows that our model is able to replicate LBBB activation metrics as well as acute electrical response to BVP and CSP. The ECG imaging data used to validate the model were collected from a different patient group to the cohort that we used to build the anatomical models. This means that the model for each patient was not individually fitted to data collected from that specific patient. Rather, we used the ECG imaging data to compare the data-derived and simulated activation metrics during LBBB and in response to different pacing modalities in the two cohorts. This showed that the model replicated measured baseline activation metrics and biventricular and LV electrical response to different pacing modalities.

Our model assumes perfect delivery of selective LBBP, while purely selective capture is difficult to achieve in clinical practice. Therefore, our results might overestimate benefits induced by LBBP and LOT-CRT. Furthermore, the clinical data we used to validate the model prediction did not include electrical synchrony induced by LOT-RT, meaning that these model predictions could not be validated. While we compared how the optimal VVD changed depending on the type of capture (selective, non-selective, or purely myocardial), we did not explicitly investigate the effects of adding an LV epicardial lead compared with LBBP lead repositioning to achieve better capture. Although these factors could be accounted with further *in silico* trials, they were outside the scope of this study. Our model did not account for LV septal scar, which has been shown to attenuate response to LBBAP.^31^ With the modelling framework used in this study, we have previously shown that LBBP is not effective in the presence of extensive LV septal scar and if the Purkinje fibres overlaying with the scar are not conductive.^12^ In these patients, LOT-CRT should not be the pacing modality of choice, due to the lack of response to LBBP.

When simulating BVP, we paced the LV epicardium at the latest activated area, without restricting to a realistic coronary sinus anatomy. Therefore, the optimal pacing location we identified might not be achievable in clinical practice. Coronary sinus anatomy segmentation was however not available for these patients. Furthermore, the validation shows that we are able to replicate LV and biventricular activation times shortening achieved with BVP measured *in vivo*.

Despite its limitations, this computational study succeeds in testing different VVDs for LOT-CRT and BVP in the presence of different LV conduction substrates. However, while our results suggest that the optimal VVD for LOT-CRT depends on the underlying LV conduction substrate, these needs to be reproduced in a clinical study to ensure clinical relevance of our findings.

## Conclusions

Left bundle branch pacing–optimized cardiac resynchronization therapy is a promising pacing modality for patients in whom delayed LV activation times cannot be completely corrected with CSP. Our modelling results show that the optimal VVD for LOT-CRT depends on the type and severity of the LV conduction substrate causing prolonged LV activation, highlighting the importance of VVD optimization for LOT-CRT delivery. This *in silico* study provides the first guidance on VVD programming for LOT-CRT in different patient groups.

## Supplementary Material

euaf089_Supplementary_Data

## Data Availability

The data underlying this article will be shared on reasonable request to the corresponding author. The cohort of heart failure anatomical models is publicly available on Zenodo (https://zenodo.org/records/3890034).
