# Supplementary material for: An in silico guide for ventriculo-ventricular delay programming for left bundle branch-optimized cardiac resynchronization therapy
Source: Europace. 2025 May 21;27(5):euaf089. doi: 10.1093/europace/euaf089 (PMC12092916; doi:10.1093/europace/euaf089)
Supplement: euaf089_Supplementary_Data [file euaf089_supplementary_data.docx]

# Supplement

## 1 Model validation

**Clinical data**

The models results have been validated on real world clinical study data from patients undergoing cardiac resynchronisation therapy at Hammersmith Hospital, London, UK.^1^ The validation was focused on the left bundle branch block (LBBB) model because this group of patients was the focus of the study. Therefore, only the data collected from LBBB patients were considered in this analysis (N=23). Electrocardiographic imaging (ECGi) was performed to measure local activation times in the ventricles at baseline (LBBB) and during biventricular pacing (BVP), His bundle pacing (HBP) and left bundle branch pacing (LBBP). Not all three pacing modalities were successfully carried out in all patients, resulting in the following patient numbers for each pacing modality:

- BVP: N=13
- HBP where LBBB was successfully corrected: N=20
- LBBP where LBBB was successfully corrected: N=21. Patients where LBBP resulted in fusion with the right bundle branch (RBB) activation were N=9

In this clinical study, the following metrics were used to compute response to pacing:

- Shortest time taken to activate 95% of the ventricles (BIVAT95)
- Shortest time taken to activate 95% of the left ventricle (LVAT95)

For the baseline activation, we also considered the total ventricular and left ventricular activation times (TAT and LVTAT).

Since the ECGi imaging data only accounted for the epicardial activation, we extracted the ventricular epicardium from the 24 anatomical models we used for the simulations. The epicardial activation during baseline (LBBB), BVP, HBP and LBBP was then used to compute response to pacing as the reduction in BIVAT95 and LVAT95 from baseline (to match the ECGi data). For LBBP, we compared LBBP simulated without atrioventricular delay optimisation against the clinical data for all patients where LBBP was successfully performed (N=21). We also compared LBBP simulated with optimised atrioventricular delay against the clinical data for patients where LBBP resulted in fusion with right bundle branch intrinsic activation (N=9).

To compare the clinical measurements and the simulations, we established whether the variables were normally distributed with a Shapiro-Wilk test with a level of significance of 0.05. We then used a two-sided t-test and a Mann-Whitney U test if the variables were normally distributed or not, respectively. We considered a statistically significant difference if the P-value was below 0.05, corrected for multiple comparisons with a Bonferroni correction (N=10 comparisons in total).

The simulated baseline activation resulted in similar activation metrics to the clinical data (Figure 1). The simulated TAT and LVTAT were 122.7 ± 12.4 ms and 121.2 ± 12.3 ms, respectively (vs clinically measured TAT 126.1 ± 19.4 ms, P=0.7279; LVTAT: 119.5 ± 20.2 ms, P=0.2234). This shows that the model is able to replicate baseline LBBB metrics.

The reduction in BIVAT95 and LVAT95 following BVP (Figure 2), HBP (Figure 3) and LBBP (Figure 4 and 5) were also similar between the simulations and the clinical data. In Table 1, we show that all comparisons apart from one resulted in non-statistically significant differences after Bonferroni correction. The only comparison that remained statistically significant was for the reduction in BIVAT95 following LBBP with atrioventricular delay optimisation, where the model slightly overestimated the electrical response (clinical: -41.8 ± 8.9 ms vs model: -53.4 ± 7.7 ms, P=0.0011, corrected P=0.011). This could be due to differences in the AV delay optimisation procedure during the clinical data acquisition and in the model.


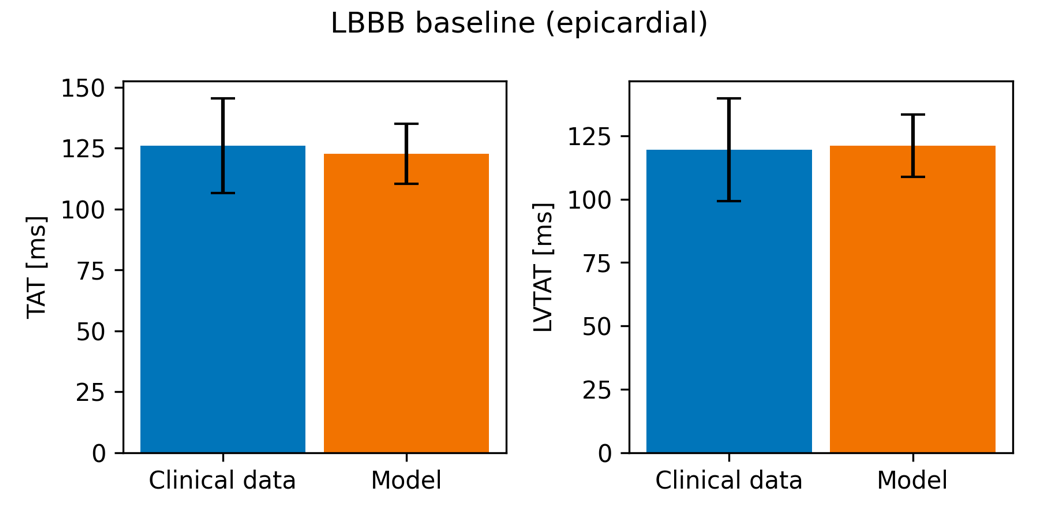


**Figure 1 Baseline LBBB epicardial simulations validation:** the mean simulated (orange) TAT (left) and LVTAT (right) were compared to the clinically measured TAT and LVAT (blue). The bars show ± one standard deviation.

**Table 1 Validation of the simulated epicardial electrical response against the clinical data.** For the comparisons that resulted in statistically differences without Bonferroni correctlon, we also reported the corrected P-value for 10 comparisons. * indicates the statistically significance differences after Bonferroni correction.

|  | **Clinical** | **Predicted epicardial** | **P-value** | **Corrected P-value** |
| --- | --- | --- | --- | --- |
| **Reduction in BIVAT95** | | | | |
| **BVP** | -19.7 ± 31.1 ms | -13.1 ± 4.4 ms | P=0.4791 |  |
| **HBP** | -46.9 ± 18.1 ms | -57.1 ± 7.2 ms | P=0.0312 | P=0.312 |
| **LBBP (no optimised AVD)** | -36.2 ± 16.2 ms | -34.3 ± 11.6 ms | P=0.6763 |  |
| **LBBP (optimised AVD)** | -41.8 ± 8.9 ms | -53.4 ± 7.7 ms | P=0.0011 | P=0.011* |
| **Reduction in LVAT95** | | | | |
| **BVP** | -12.0 ± 36.1 ms | -7.4 ± 5.8 ms | P=0.6705 |  |
| **HBP** | -44.3 ± 19.9 ms | -50.4 ± 8.5 ms | P=0.2225 |  |
| **LBBP (no optimised AVD)** | -44.5 ± 19.8 ms | -51.1 ± 9.1 ms | P=0.1796 |  |
| **LBBP (optimised AVD)** | -55.0 ± 15.8 ms | -49.1 ± 8.1 ms | P=0.3333 |  |

**
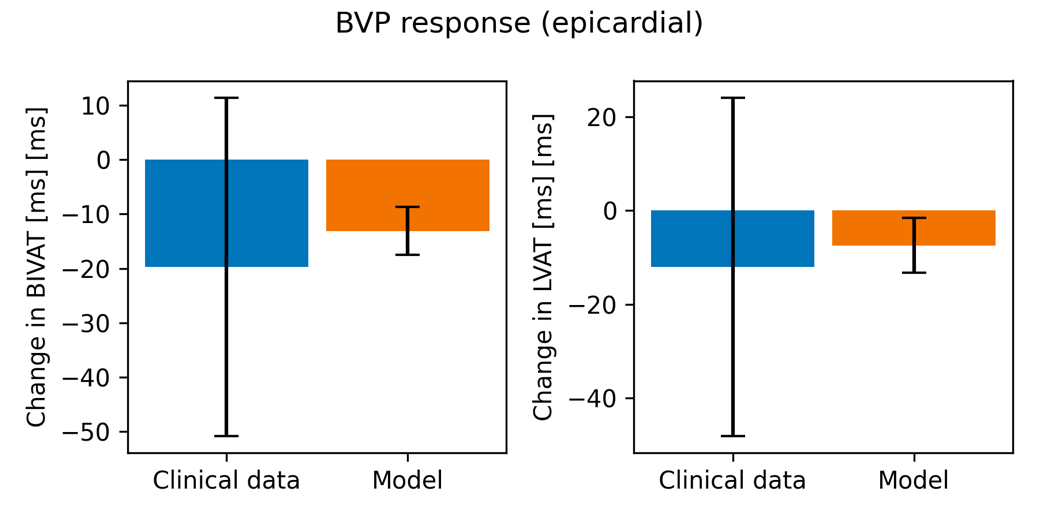
**

**Figure 2 Validation of the epicardial electrical response predictions during BVP:** the mean simulated (orange) reduction in ventricular activation times (left) and LV activation times (right) following BVP were compared to the clinically electrical response through ECGi. The bars show ± one standard deviation.

***
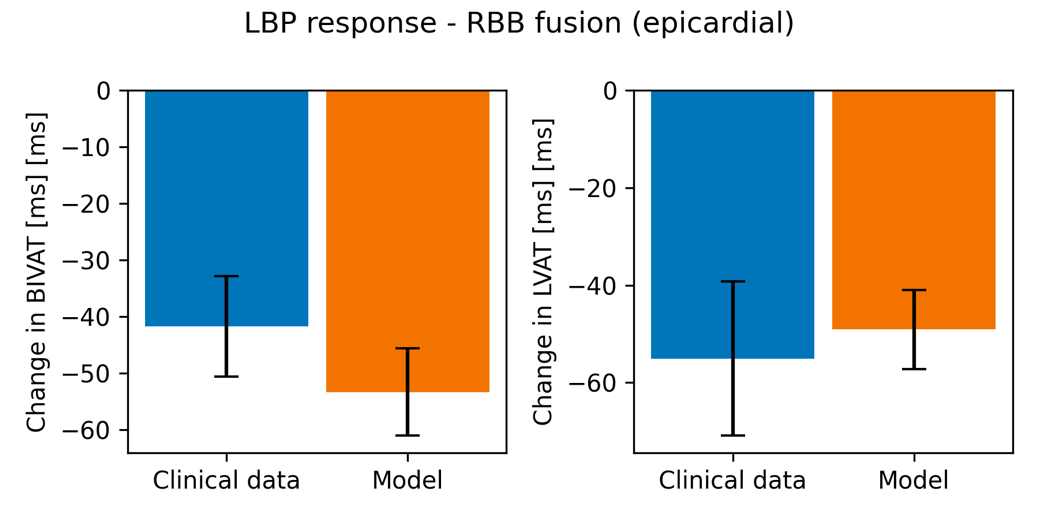
*
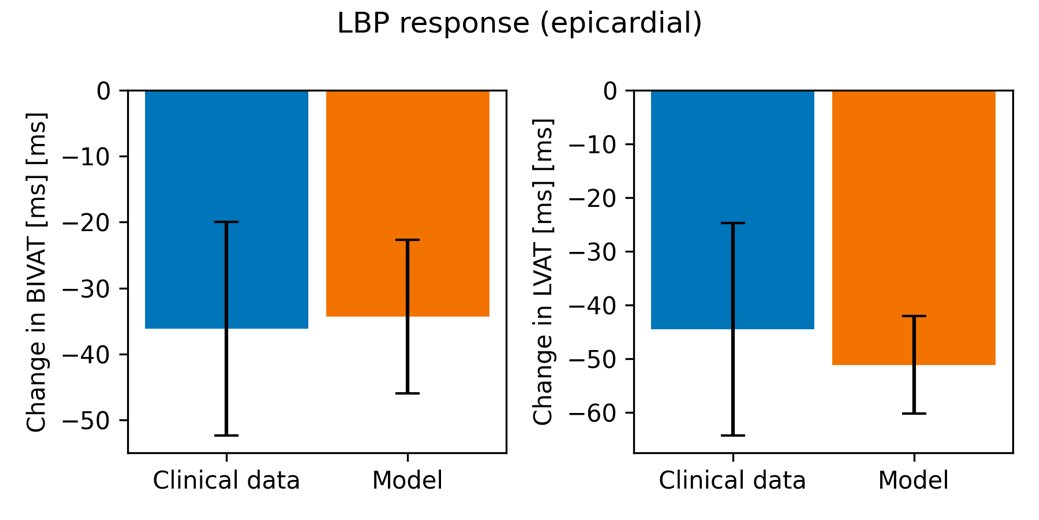

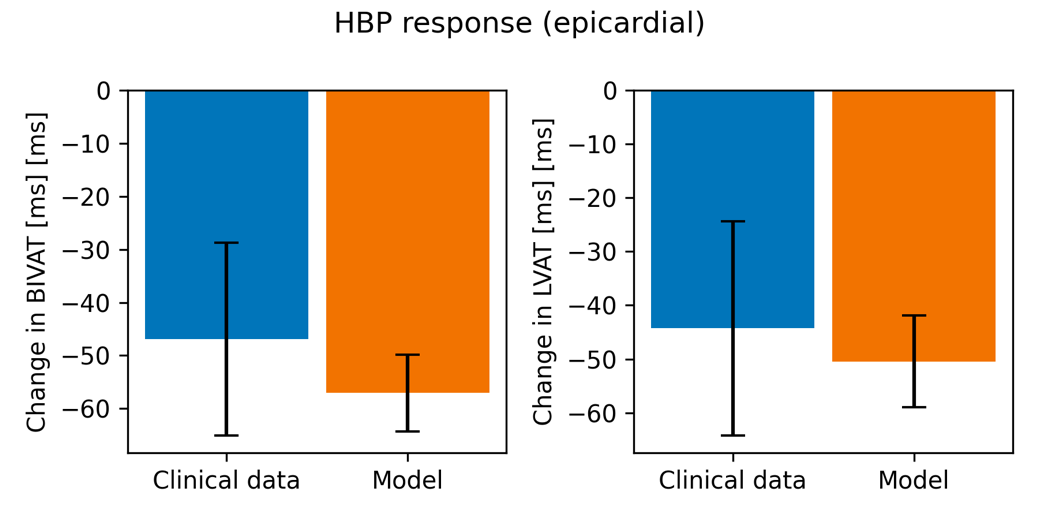
**.

**Figure 5 Validation of the epicardial electrical response predictions during LBBP (with atrioventricular delay optimisation):** the mean simulated (orange) reduction in ventricular activation times (left) and LV activation times (right) following LBBP performed without atrioventricular delay optimisation were compared to the clinically electrical response through ECGi. The bars show ± one standard deviation. In this case, we considered clinical data collected from all patients where LBBP resulted in fusion with RBB activation (N=9).

**Figure 4 Validation of the epicardial electrical response predictions during LBBP (without atrioventricular delay optimisation):** the mean simulated (orange) reduction in ventricular activation times (left) and LV activation times (right) following LBBP performed without atrioventricular delay optimisation were compared to the clinically electrical response through ECGi. The bars show ± one standard deviation. In this case, we considered clinical data collected from all patients where LBBP was performed successfully (N=21).

**Figure 3 Validation of the epicardial electrical response predictions during HBP:** the mean simulated (orange) reduction in ventricular activation times (left) and LV activation times (right) following HBP were compared to the clinically electrical response through ECGi. The bars show ± one standard deviation.

This comprehensive validation shows that the baseline LBBB model operates within similar activation times ranges as the clinical data, showing that the activation metrics simulated by the model is in agreement with values measured in LBBB patients. We also show that the model is able to replicate LV and biventricular response to BVP, HBP and LBBP. We can therefore conclude that the model is suitable to study changes in ventricular activation during different pacing modalities.

# 2 Additional results

In the following sections, we show additional results for the following scenarios:

- ***Proximal LBBB without any underlying conduction substrate*** to quantify the contribution of LV epicardial pacing towards LV synchrony during LOT-CRT in patients with no LV conduction disease apart from proximal conduction block
- ***His-optimised CRT (HOT-CRT)*** to test whether the optimal VVD is different between HOT-CRT vs LOT-CRT
- ***LOT-CRT with optimised atrioventricular delay (AVD)*** to test whether AVD optimisation changed the optimal VVD for LOT-CRT
- ***Scar in the LV lateral wall*** to quantify the optimal VVD in the presence of regional rather than uniform LV myocardial conduction slowing.

## Proximal left bundle branch block with no underlying substrate

In patients with purely proximal LBBB, LOT-CRT would not be attempted, as LBBP alone is able to normalise LV activation times. However, to quantify the contribution of LV epicardial pacing towards LV synchrony, we performed simulations with LOT-CRT in the presence of proximally LBBB with otherwise healthy conduction (Figure 6, left). For comparison, we also simulated BVP with VVD ranging between -100 ms and +100 ms. Our results show that when the left bundle is paced ahead of the LV epicardium (VVD ≤ -80ms), the LV epicardial stimulus has no effect as the whole LV has already activated, therefore leading to the same LVAT95 for VVDs between -100 ms and -80 ms (Figure 6, left). The optimal VVD for LOT-CRT was -10.0 ± 5.0 ms and led to an LVAT95 of 46.3 ± 3.9 ms. These LVAT95 were only marginally shorter than those obtained with LBBP alone (52.2 ± 5.1 ms). BVP resulted in the shortest LVAT95 with a slightly negative VVD of -7.5 ± 23.0 ms (Figure 6, right), although all near-simultaneous VVDs achieve similar LVAT95 (VVD -20 ms: 76.4 ± 7.1 ms; VVD +20 ms: 79.1 ± 8.6 ms). These results indicate that with proximal LBBB alone with no other conduction disorders, LV epicardial pacing provides limited benefits to LV synchrony, and that near simultaneous BVP leads to the shortest LVAT95.


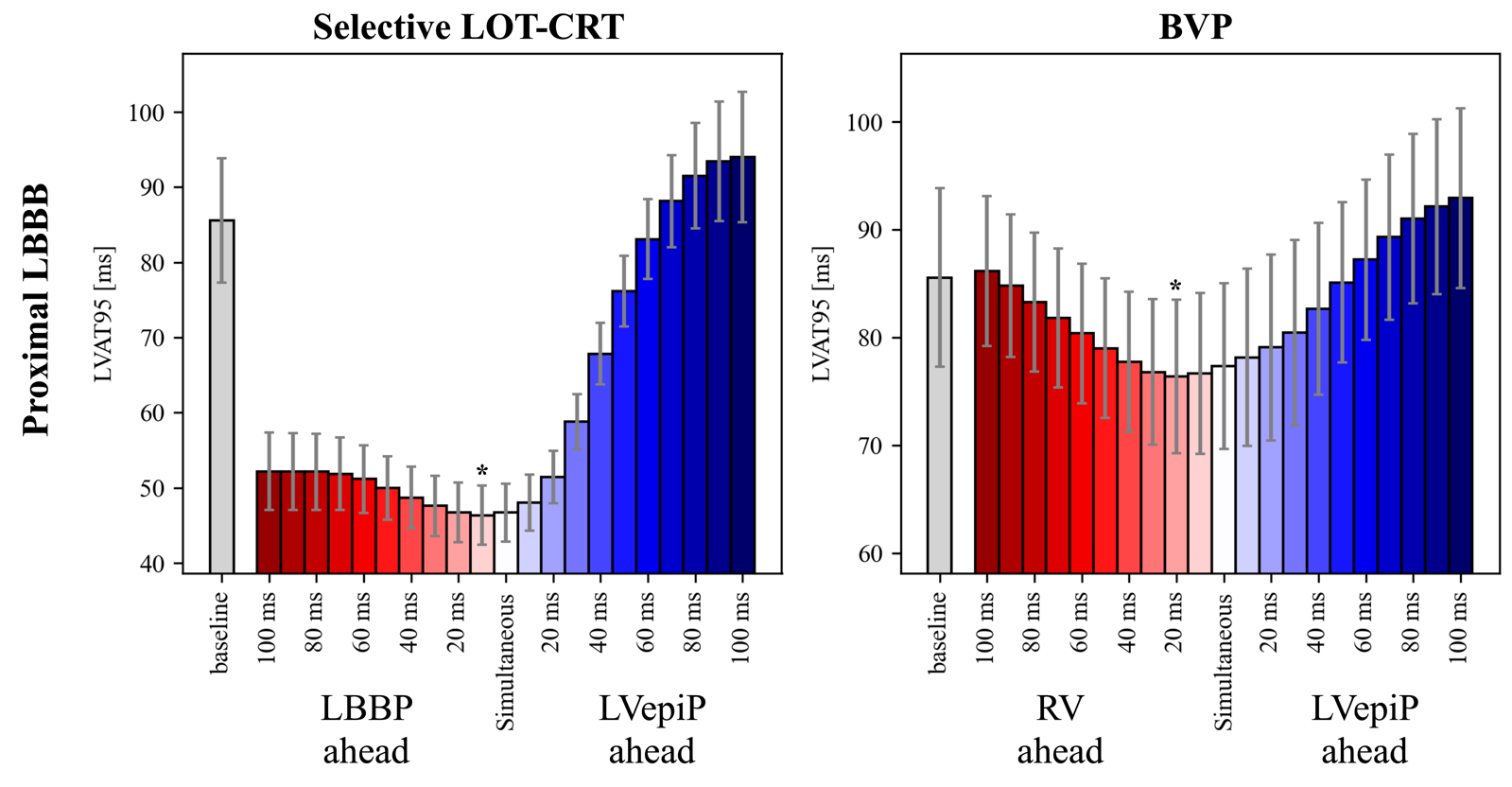


**Figure 6 Response to LOT-CRT for different VVDs for proximal LBBB and no other LV conduction substrate.** The figures show the LVAT95 for proximal LBBB with otherwise healthy conduction for LOT-CRT (left) and BVP (right). All results are shown for VVDs ranging from -100 ms (LBBP/RV ahead) to +100 ms (LVepiP ahead). The grey bars and the stars indicate the LVAT95 at baseline and the best VVD on average, respectively, while the error bars indicate ± the standard deviation

## HOT-CRT compared to LOT-CRT

While LOT-CRT combines LBBP and LV epicardial pacing, HOT-CRT is performed by pacing the His bundle pacing and the LV epicardium. Because HOT-CRT is becoming increasingly popular as a pacing modality for patients with residual delayed LV activation following conduction system pacing, we tested whether the optimal VVD for HOT-CRT and LOT-CRT was different. To this aim, we ran simulations with HOT-CRT for different VVDs in the presence of proximal LBBB combined with diffuse LV conduction system disease (40% slow LV His-Purkinje) or diffuse LV intramyocardial delay (40% slow LV myocardium) and compared the results with LOT-CRT (Figure 7). HOT-CRT (Figure 7, top) and LOT-CRT (Figure 7, bottom) resulted in similar VVDs. The optimal VVD for HOT-CRT in the presence of LBBB and diffuse LV conduction system disease was -42.5 ± 7.2 ms compared to -42.5 ± 6.6 ms with LOT-CRT, meaning that the conduction system is stimulated early to compensate for the slow LV His-Purkinje system. In the presence of LBBB and LV intramyocardial delay, the optimal VVD for HOT-CRT and LOT-CRT was 21.2 ± 9.3 ms and 23.3 ± 8.5 ms, respectively, indicating the LV epicardium is paced ahead to compensate for the slow LV tissue. The optimal VVD for HOT-CRT and LOT-CRT is similar, demonstrating that our results about VVD optimisation are valid for HOT-CRT as well as LOT-CRT.


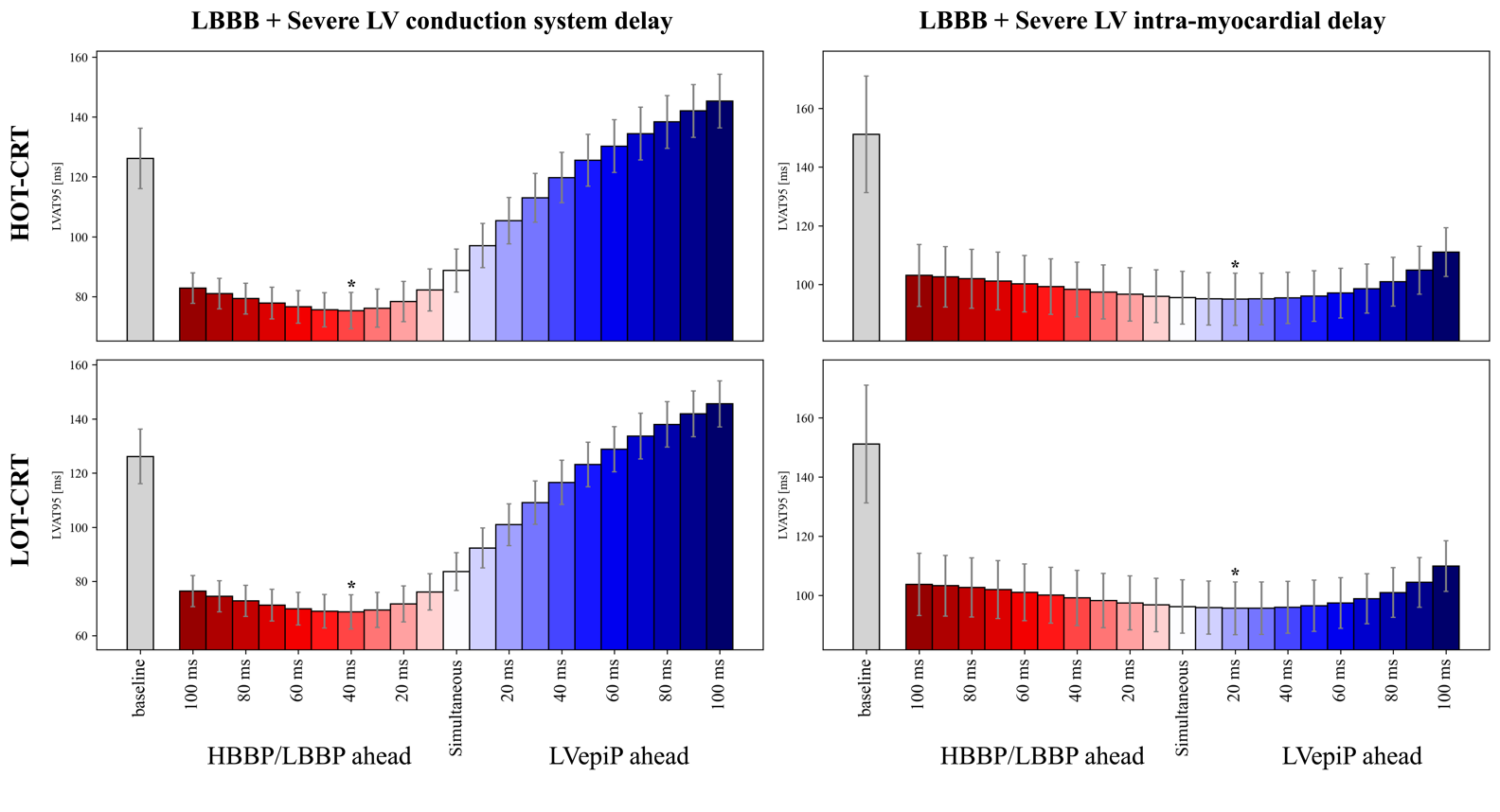


**Figure 7 Response to HOT-CRT and LOT-CRT for different VVDs for different LV conduction substrates.** The top and bottom rows show the LVAT95 for proximal LBBB combined with different LV conduction system disorders for HOT-CRT and LOT-CRT, respectively. Left: proximal LBBB combined with severe LV conduction system delay, simulated as LV myocardial CV slowed to 40% of healthy CV; right: proximal LBBB combined with severe LV diffuse intra-myocardial delay, simulated as LV myocardium CV slowed to 40% of healthy CV. All results are shown for VVDs ranging from -100 ms (HBBP/LBBP ahead) to +100 ms (LVepiP ahead). The grey bars and the stars indicate the LVAT95 at baseline and the best VVD on average, respectively, while the error bars indicate ± the standard deviation.

## LOT-CRT with optimised atrioventricular delay

LOT-CRT aims at shortening LV activation times and to correct residual delayed LV activation that cannot be corrected with LBBP alone. Clinical^2^ and computational^3^ studies have shown that LBBP leads to short LV activation times, but it can prolong right ventricular (RV) activation times that can be attenuated by optimising the atrioventricular delay (AVD) in patients with viable intrinsic right bundle conduction. To test if the optimal VVD for LOT-CRT changed if performed with LBBP with or without optimised AVD, we ran simulations with LBBP combined with a His stimulus to simulate the intrinsic activation travelling down from the atria along the right bundle. We changed the delay between LBBP and the His stimulus between -50 ms (LBBP ahead) and +50 ms (intrinsic ahead) to simulate AVD optimisation. The optimal AVD was selected for each patient as the delay leading to the shortest biventricular activation times (BIVAT90). LOT-CRT with optimised AVD was simulated by adding an LV epicardial stimulus, and VVD was ranged between -100 ms (LBBP ahead) and +100 ms (LV epicardial pacing ahead).

The optimal VVD for LOT-CRT with optimised AVD (LOT-CRT-AVD) was similar to the optimal VVD obtained with LOT-CRT without AVD optimisation. In the presence of proximal LBBB and mild or severe LV conduction system delay, the optimal VVD for LOT-CRT-AVD was -24.2 ± 6.4 ms and -42.5 ± 6.6 ms, respectively (compared to LOT-CRT without AVD optimisation -24.2 ± 6.4 ms and -42.5 ± 6.6 ms, Figure 8 first two columns). Similarly, with proximal LBBB and mild or severe intramyocardial delay, the optimal VVD for LOT-CRT-AVD was 5.4 ± 6.4 ms and 23.3 ± 9.5 ms, respectively (compared to LOT-CRT without AVD optimisation 5.4 ± 6.4 ms and 23.3 ± 8.5 ms, Figure 8 last two columns). Figure 9 shows that the AVD optimisation only affects RV activation times, while the LV activation times are shortened by LBBP and LV epicardial pacing. These results demonstrate that AVD optimisation can be used to optimise inter-ventricular synchrony in patients with viable intrinsic right bundle branch conduction, while leaving LV activation times unaffected.

**
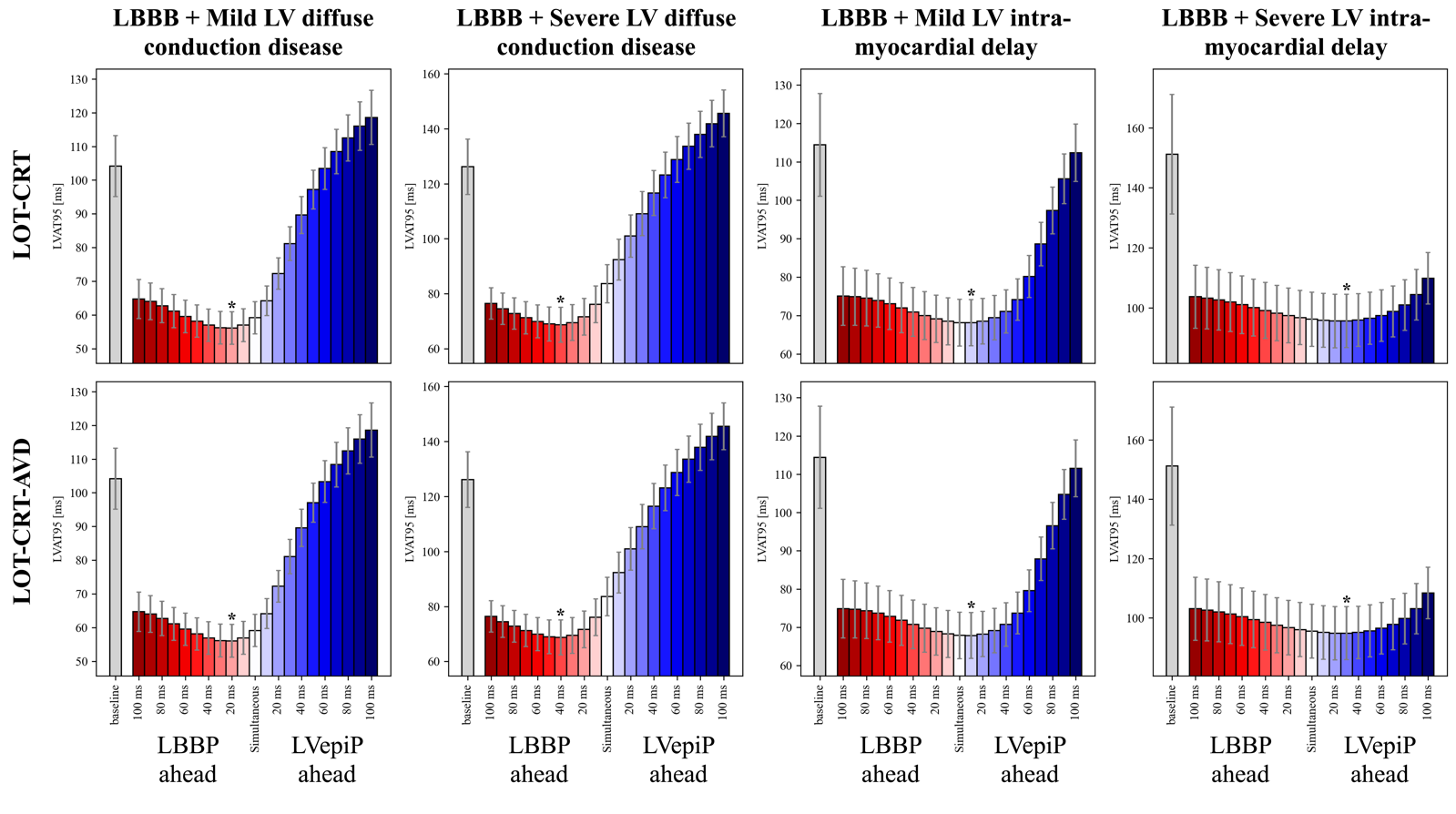
**

**Figure 8 Response to LOT-CRT with and without atrioventricular delay optimisation for different VVDs for different LV conduction system disorders.** Mean LVAT95 is shown as a function of VVDs during LOT-CRT without (top row) and with atrioventricular delay optimisation (bottom row). Different columns represent different conduction substrates in combination with proximal LBBB. From left to right: mild and severe LV conduction system delay, simulated as LV His-Purkinje system CV slowed to 60% and 40% of healthy CV; mild and severe LV intra-myocardial delay, simulated as LV myocardium CV slowed to 60% and 40% of healthy CV. All results are shown for VVDs ranging from -100 ms (LBBP or RV pacing ahead) to +100 ms (LVepiP ahead). The grey bars and the stars indicate the LVAT95 at baseline and the best VVD on average, respectively, while the error bars indicate ± the standard deviation.


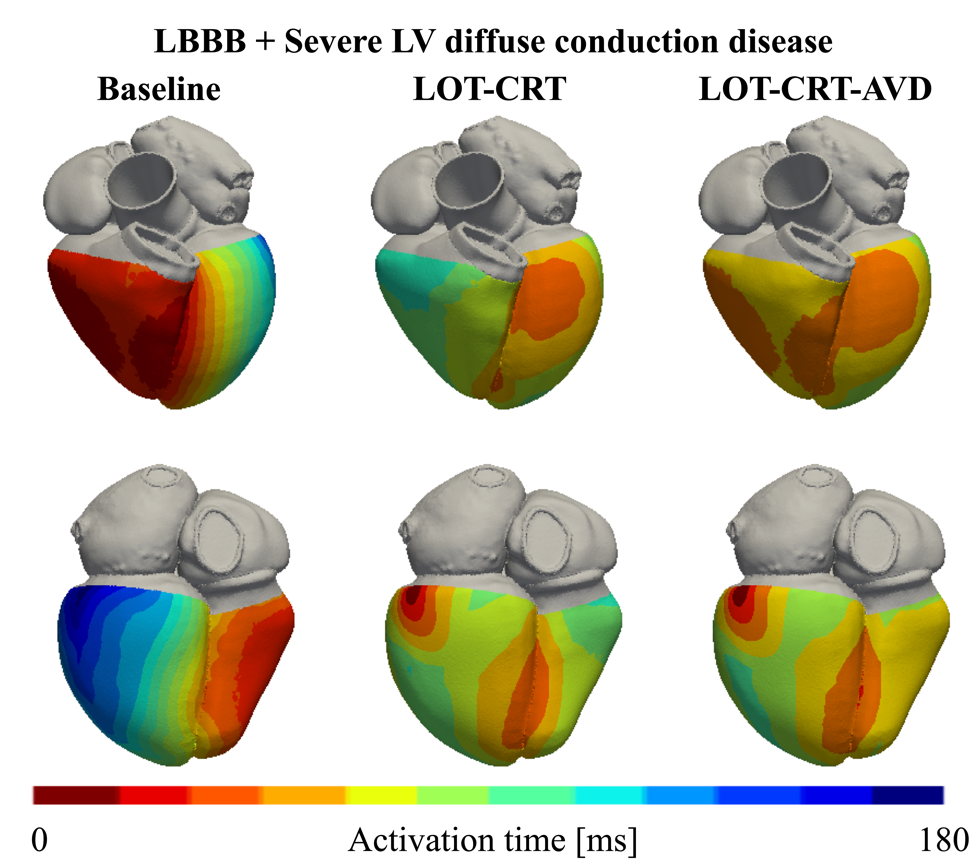


**Figure 9 Simulated activation maps during LOT-CRT with and without AVD optimisation**. The simulated ventricular activation times are shown for LBBB combined with severe LV diffuse conduction disease (LV His-Purkine system with CV set to be 40% of healthy CV). The top and bottom rows show an anterior and a posterior view, respectively. From left to right: baseline, LOT-CRT without AVD optimisation, LOT-CRT with AVD optimisation. Red and blue areas show early and late activated regions, respectively.

# Lateral scar in the left ventricular free wall

In the manuscript, we simulated intra-myocardial delay as a uniformly slow conducting LV myocardium. However, in some patients, slow LV conduction may be regional due to the presence of scar. We included this scenario by mapping a LV scar geometry segmented from late-gadolinium enhanced MRI from a publicly available LV anatomical model from Costa et al^4^ onto all our twenty-four whole-heart models as described previously^5^. Figure 10 (left) shows the scar core and border zone mapped onto one of the models. We then simulated four scenarios:

1. proximal LBBB and non-conductive LV lateral wall scar and scarred Purkinje (that is, the Purkinje overlapping with the scar core)
2. proximal LBBB and slow conductive LV lateral wall scar (conduction velocity set to 40% of healthy myocardium) and scarred Purkinje (conduction velocity set to 40% of healthy His-Purkinje)
3. Healthy proximal conduction and non-conductive LV lateral wall scar and scarred Purkinje
4. Healthy proximal conduction and slow conductive LV lateral wall scar (conduction velocity set to 20% of healthy myocardium) and scarred Purkinje (conduction velocity set to 20% of healthy His-Purkinje)

Unless stated otherwise, the parameters were set to the baseline model described in the manuscript. The conduction velocity in the border zone set to isotropic and 50% of the transverse conduction velocity of healthy myocardium.^4^ Figure 10 (right) summarises these four scenarios. LOT-CRT with selective capture was then simulated with different VVDs (between -100 ms and +100 ms) for these cases. To avoid pacing within the scar, the latest activated area was restricted to epicardial regions above 70% of the apico-basal direction of the ventricles using the universal ventricular coordinates^6,7^.


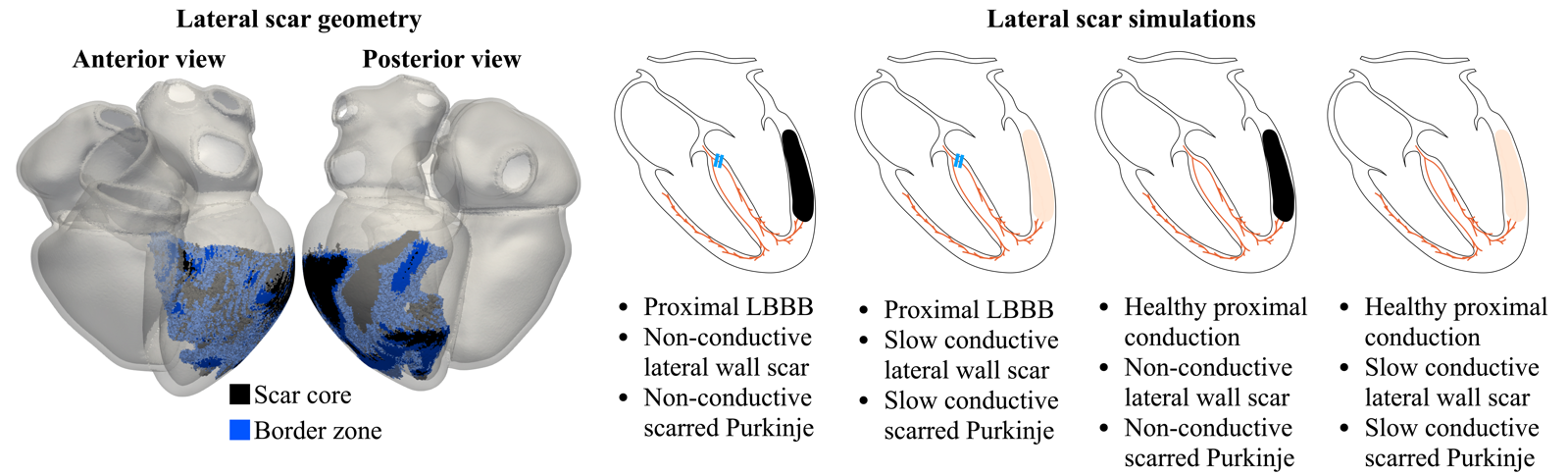


**Figure 10 Simulations with scar in the LV lateral wall.** Left: LV lateral wall scar mapped onto one of our four-chamber anatomical models, with black and blue regions indicating the scar core and the border zone, respectively. Right: simulation scenarios we considered.

The optimal VVD for LOT-CRT changes depending on the conduction properties of the scar (Figure 11). In the presence of proximal LBBB and non-conductive scar, the optimal VVD for LOT-CRT was positive (VVD: 17.5±15.9 ms). In the presence of proximal LBBB and slow conductive scar and slow scarred Purkinje, the shortest LVAT95 was achieved with a slightly negative VVD for LOT-CRT (VVD: -7.5 ± 21.3 ms) to compensate for the locally slow LV His-Purkinje below the scar. When the proximal His-Purkinje system was healthy and the scar was non-conductive, LOT-CRT resulted in the shortest LVAT95 with a slightly positive VVD (7.5±13.9 ms), while when the scar was slow and conductive, the optimal VVD was positive to compensate for the slow LV conduction in the lateral wall (VVD: 30.0 ± 21.6 ms). These results demonstrate that the optimal VVD for LOT-CRT depends not only on the presence of LV lateral wall scar, but also on the conduction properties of the scarred tissue.


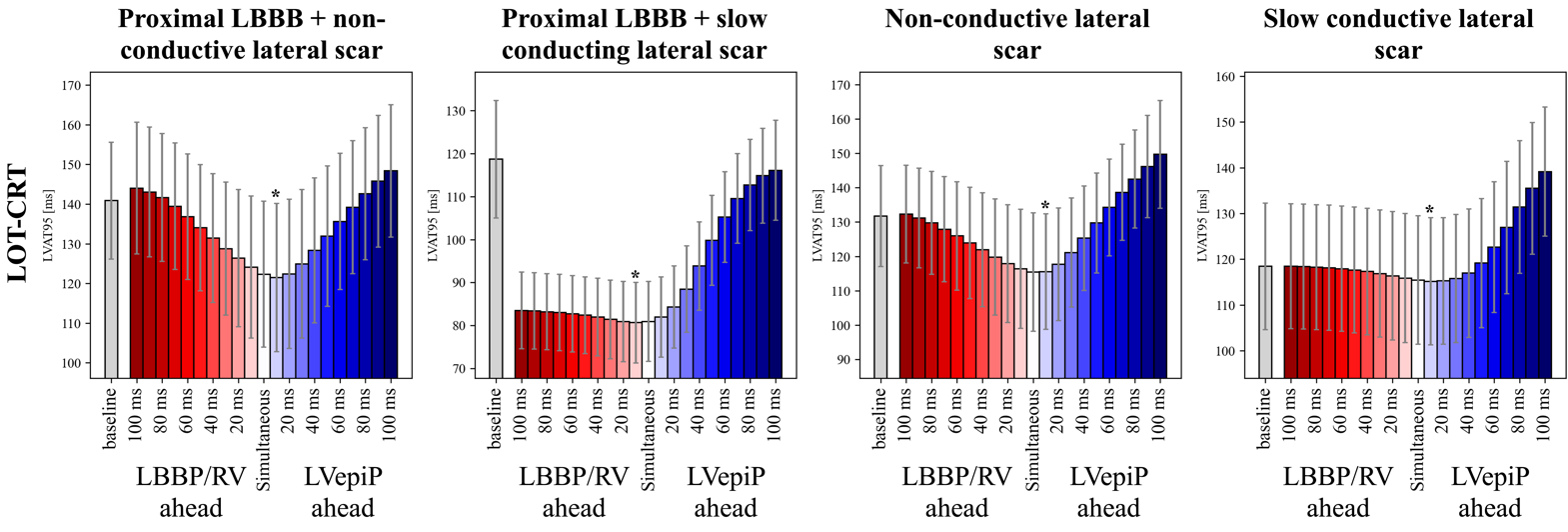


**Figure 11 Response to LOT-CRT in the presence of LV lateral wall scar.** Mean LVAT95 is shown as a function of VVDs during LOT-CRT in the presence of LV lateral wall scar. Different columns represent different conduction. From left to right: proximal LBBB in combination with non-conductive LV lateral wall scar; proximal LBBB in combination with slow conducting LV lateral wall scar; healthy proximal conduction in combination with non-conductive LV lateral wall scar; healthy proximal conduction in combination with slow conducting LV lateral wall scar. All results are shown for VVDs ranging from -100 ms (LBBP or RV pacing ahead) to +100 ms (LVepiP ahead). The grey bars and the stars indicate the LVAT95 at baseline and the best VVD on average, respectively, while the error bars indicate ± the standard deviation.

# References

1. Ali N, Arnold AD, Miyazawa AA, et al.: Comparison of methods for delivering cardiac resynchronization therapy: an acute electrical and haemodynamic within-patient comparison of left bundle branch area, His bundle, and biventricular pacing. EP Europace 2023; .

2. Lin J, Dai Y, Wang H, Li Y, Chen K, Zhang S: A comparison of left bundle branch pacing with His bundle pacing in a patient with heart failure and left bundle branch block. HeartRhythm Case Rep 2020; 6.

3. Strocchi M, Lee AWC, Neic A, et al.: His-bundle and left bundle pacing with optimized atrioventricular delay achieve superior electrical synchrony over endocardial and epicardial pacing in left bundle branch block patients. Heart Rhythm 2020; 17.

4. Mendonca Costa C, Neic A, Kerfoot E, et al.: Pacing in proximity to scar during cardiac resynchronization therapy increases local dispersion of repolarization and susceptibility to ventricular arrhythmogenesis. Heart Rhythm 2019; 16.

5. Strocchi M, Gillette K, Neic A, et al.: Effect of scar and His–Purkinje and myocardium conduction on response to conduction system pacing. J Cardiovasc Electrophysiol 2023; 34.

6. Bayer J, Prassl AJ, Pashaei A, et al.: Universal ventricular coordinates: A generic framework for describing position within the heart and transferring data. Med Image Anal 2018; 45.

7. Gillette K, Gsell MAF, Bouyssier J, et al.: Automated Framework for the Inclusion of a His–Purkinje System in Cardiac Digital Twins of Ventricular Electrophysiology. Ann Biomed Eng 2021; 49.
